# Supplementary figures and images for: Somatic nuclear mitochondrial DNA insertions are prevalent in the human brain and accumulate over time in fibroblasts
Source: PLoS Biol. 2024 Aug 22;22(8):e3002723. doi: 10.1371/journal.pbio.3002723 (PMC11340991; doi:10.1371/journal.pbio.3002723)

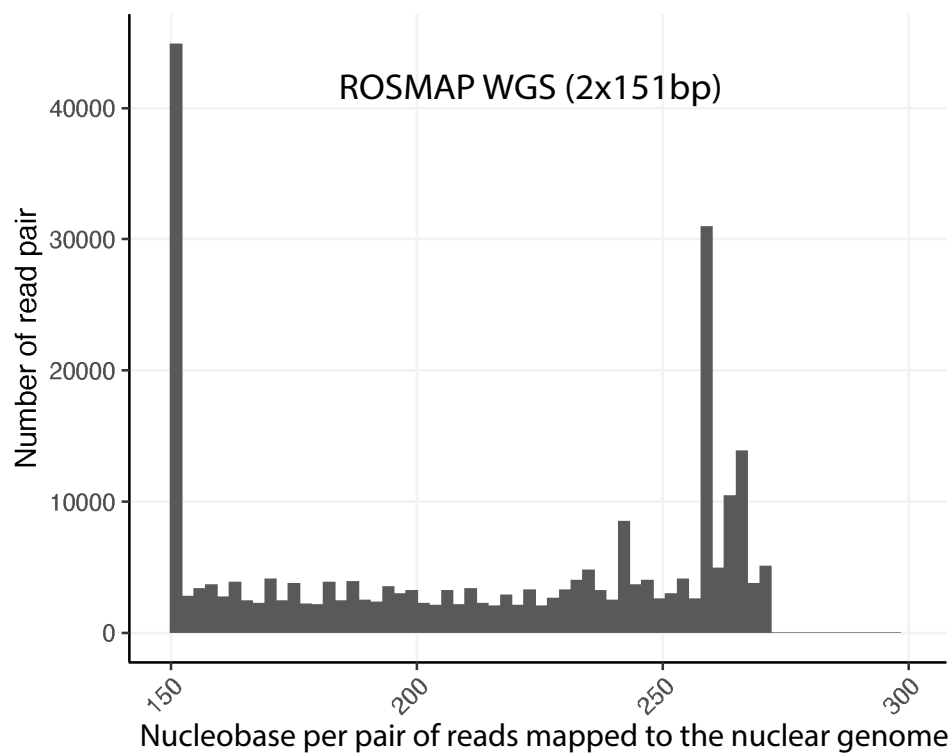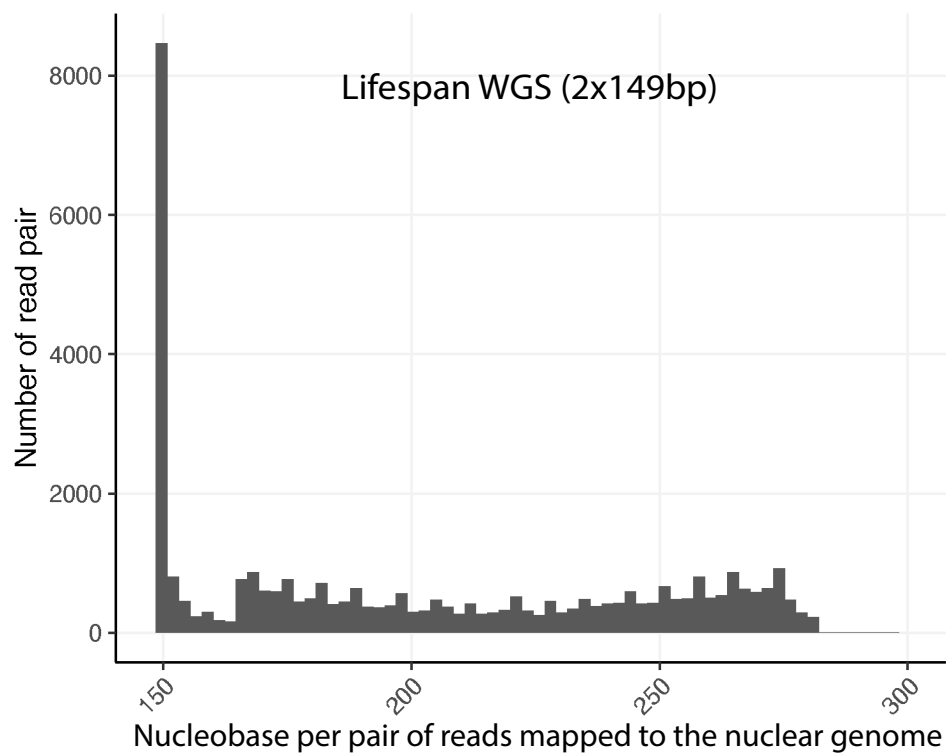

Supplement: S1 Fig — Left, 2 × 151 bp in ROSMAP and right, 2 × 149 bp in the lifespan WGS. The data underlying this figure can be found in S1 Data. (PDF) [file pbio.3002723.s007.pdf]

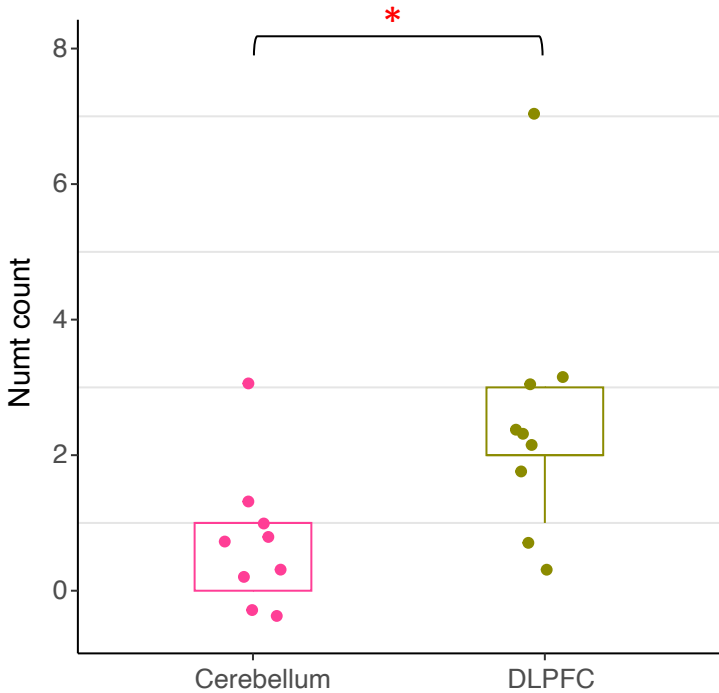

Supplement: S2 Fig — P-value = 0.033, Student’s T test, paired, two-sided. Samples are shown in jittered points. The data underlying this figure can be found in S1 Data. (PDF) [file pbio.3002723.s008.pdf]

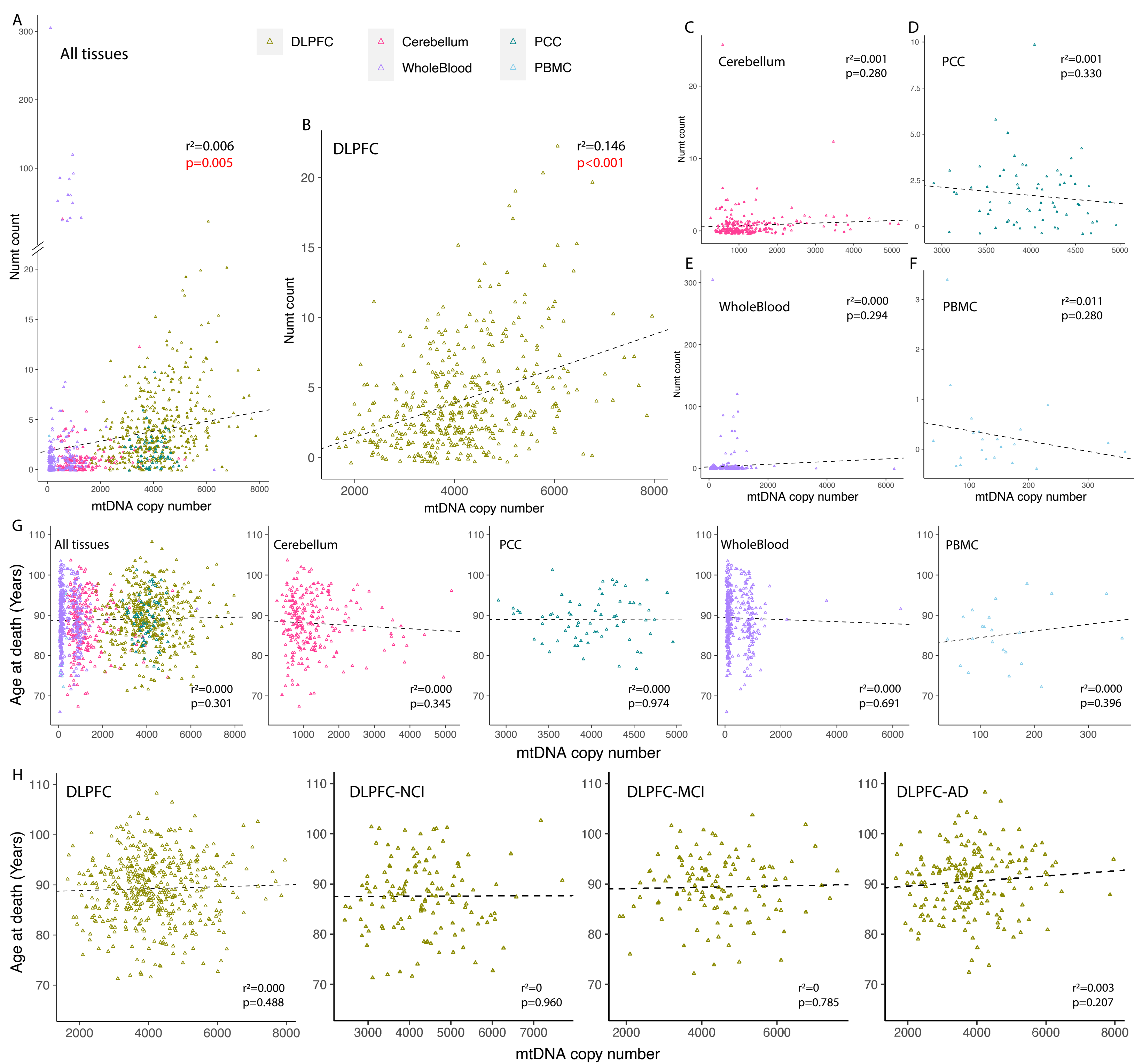

Supplement: S3 Fig — Correlation between mtDNA copy number and Numt count in all ROSMAP samples (A), DLPFC (B), cerebellum (C), PCC (D), whole blood (E), and PBMC (F), respectively. (G) Correlation between mtDNA copy number and age at death in all ROSMAP samples, cerebellum, PCC, whole blood, and PBMC, respectively. (H) Correlation between mtDNA copy number and age at death in DLPFC and 3 cognitive groups in DLPFC, respectively. r2 and p-values are calculated using standard least-squares regression models. The data underlying this figure can be found in S1 Data. (PDF) [file pbio.3002723.s009.pdf]

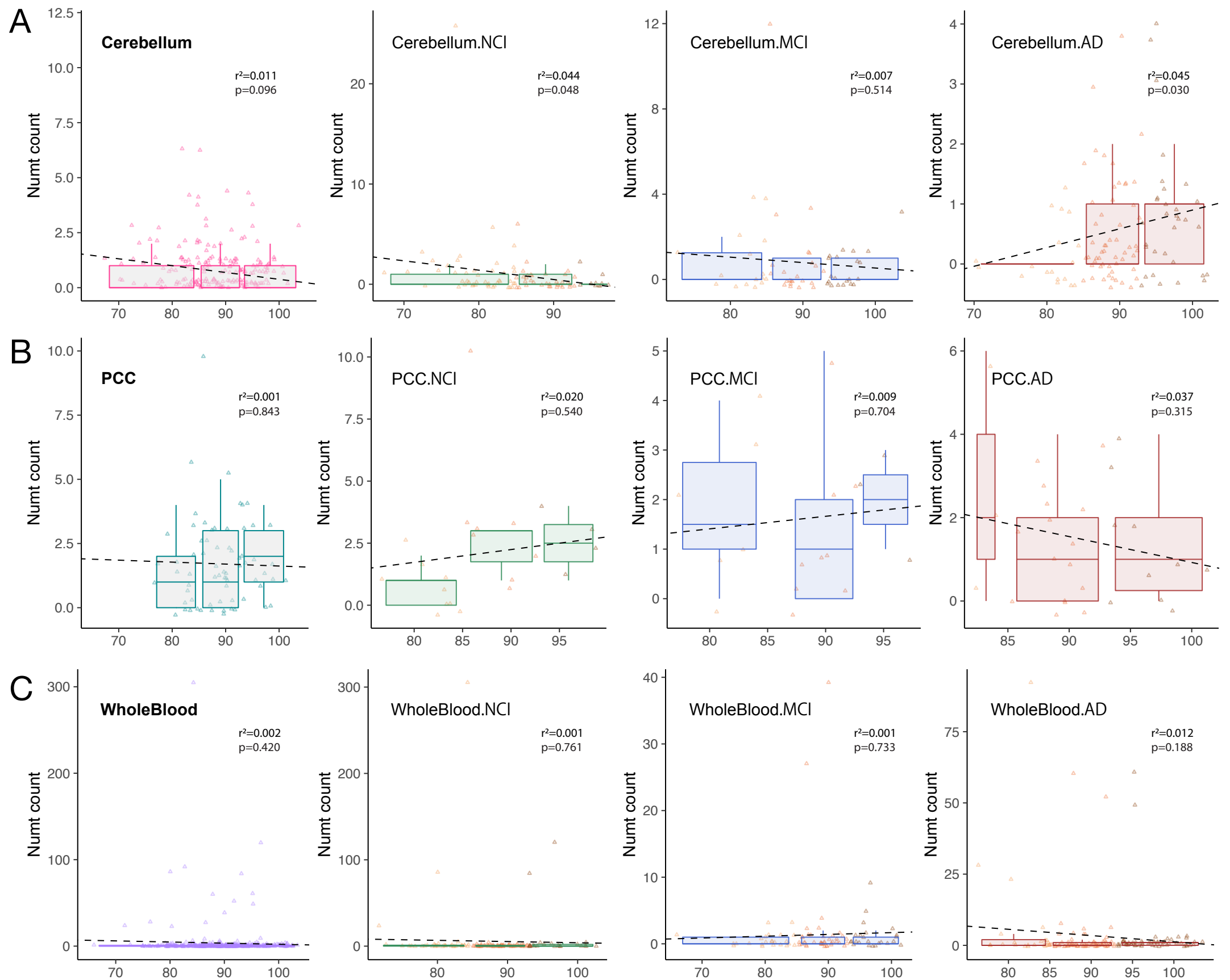

Supplement: S4 Fig — (A) Cerebellum samples correlated with age at death, stratified by cognitive diagnosis status. (B) PCC samples correlated with age at death, stratified by cognitive diagnosis status. (C) Whole-blood samples correlated with age at death, stratified by cognitive diagnosis status. Data points are colored by arbitrary age groups (see Methods) in light yellow, orange, and brown, respectively. r2 and p-values are calculated using standard least-squares regression models. (PDF) [file pbio.3002723.s010.pdf]

**A**

Numts

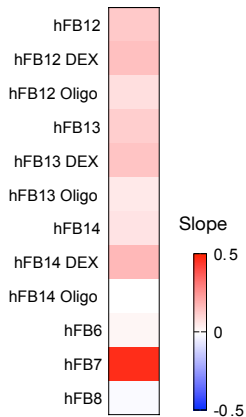

Numts(Dinumt)

**B**

LINE1 ALU SVA

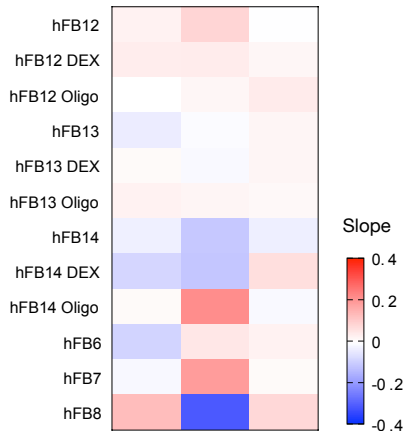

MEIs (MELT)

**C**

DEL DUP INV INS BND

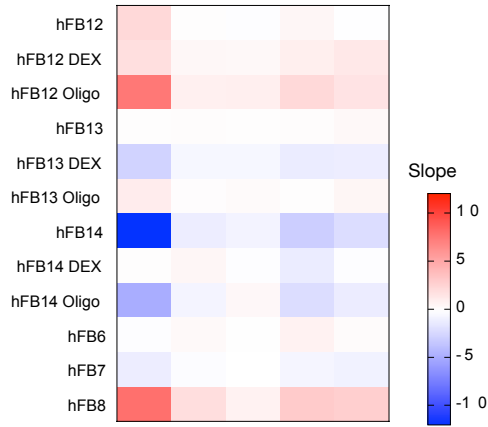

SVs (DELLY)

Supplement: S6 Fig — (A) Heatmap of slopes based on the linear regression between days cultured and the cell line-specific Numts (from Dinumt). (B) Heatmap of slopes based on the linear regression between days cultured and the cell line-specific MEIs (from MELT). (C) Heatmap of slopes based on the linear regression between days cultured and the cell line-specific SVs (from DELLY). (PDF) [file pbio.3002723.s012.pdf]

A

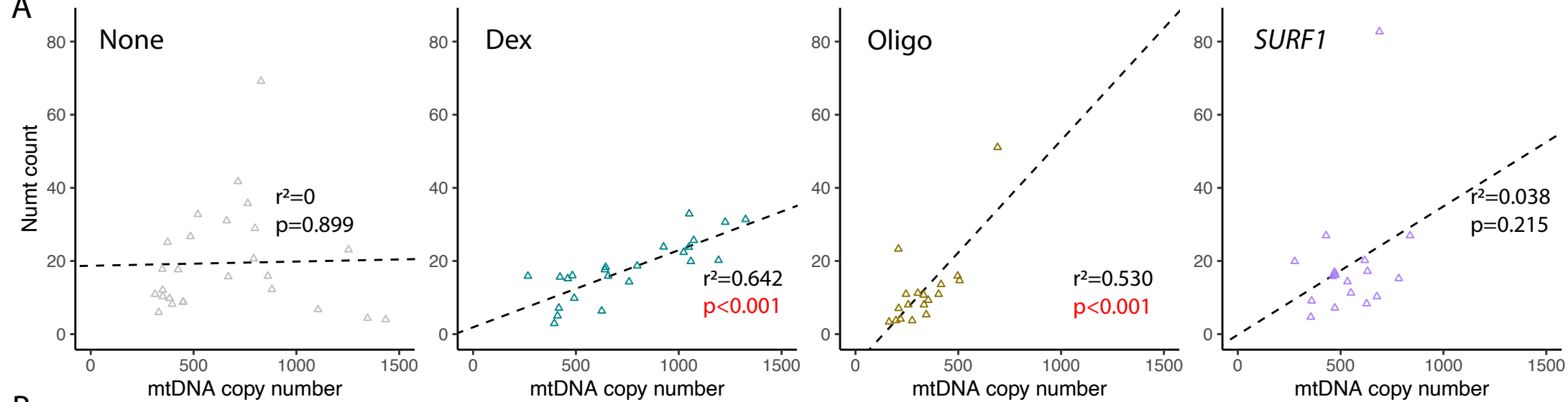

B

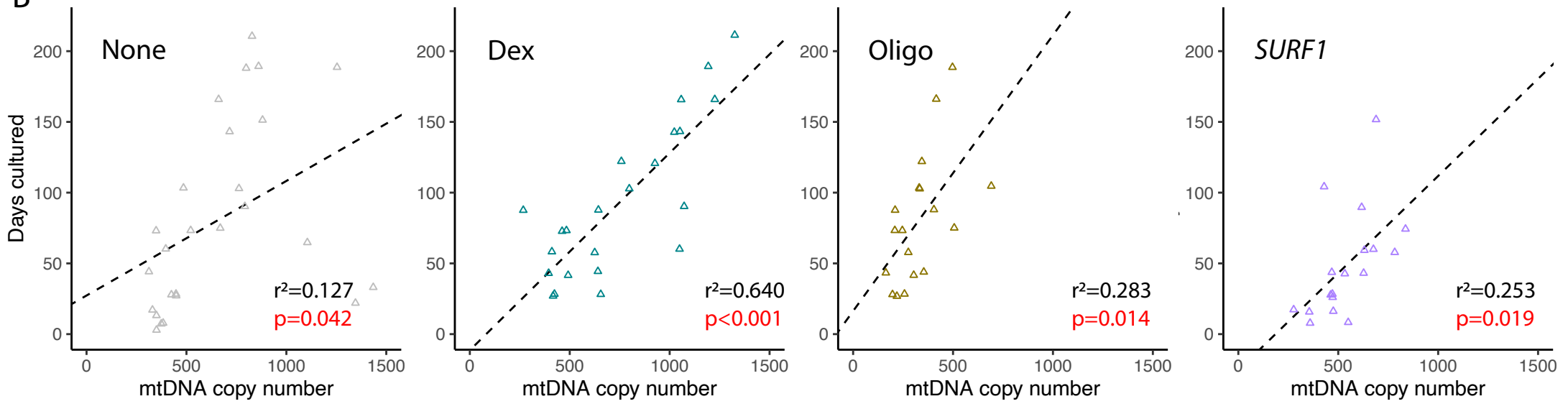

Supplement: S7 Fig — (A) Correlation between mtDNA copy number and Numt count in 3 treatment groups and SURF1 defect group, respectively. (B) Correlation between mtDNA copy number and the number of days cells were cultured (Days cultured) in 3 treatment groups and SURF1 defect group, respectively. r2 and p-values are calculated using standard least-squares regression models. The data underlying this figure can be found in S1 Data. (PDF) [file pbio.3002723.s013.pdf]

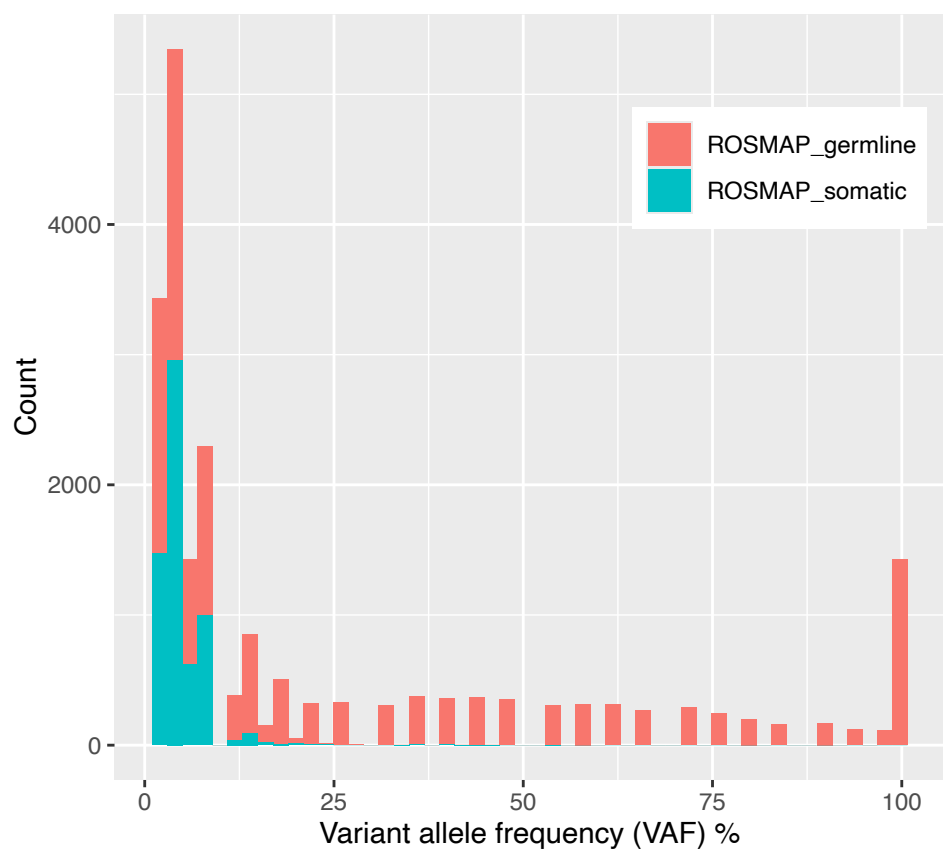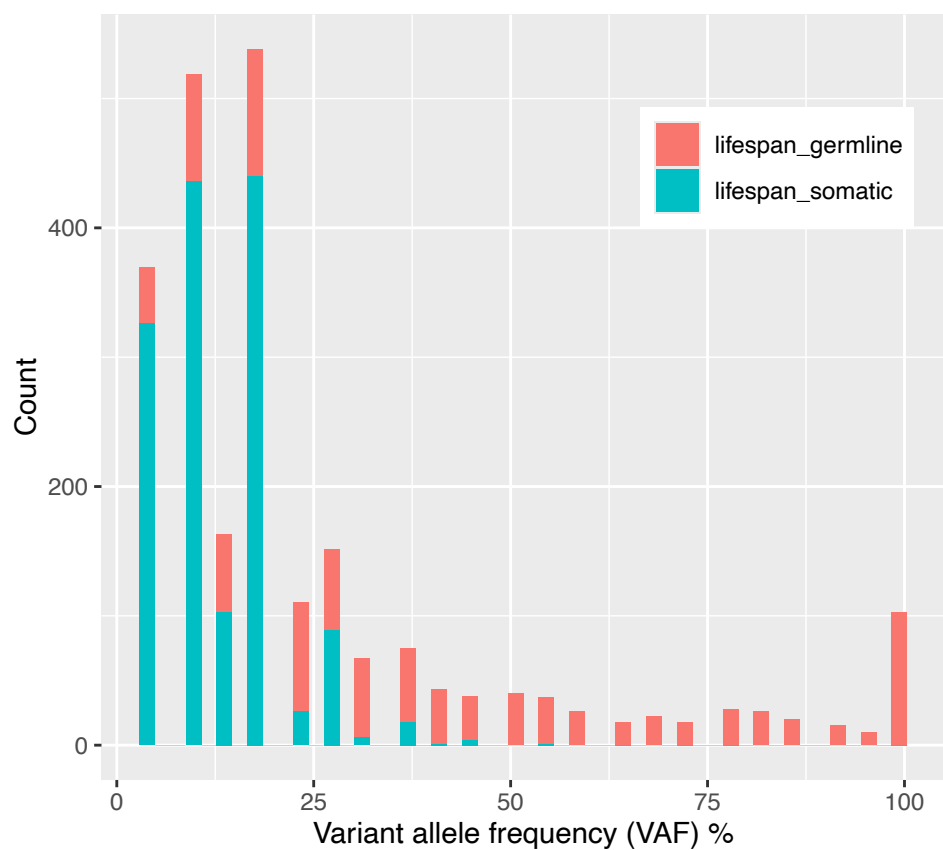

Supplement: S8 Fig — The Numts were categorized into germline ones (red), which overlapped with 1KG and Wei and colleagues callset and shared between tissues (ROSMAP, left) or cell lines (lifespan study, right), and potential somatic ones (green), which are tissue-specific (ROSMAP, left) or cell line-specific (lifespan study, right). The data underlying this figure can be found in S1 Data. (PDF) [file pbio.3002723.s014.pdf]

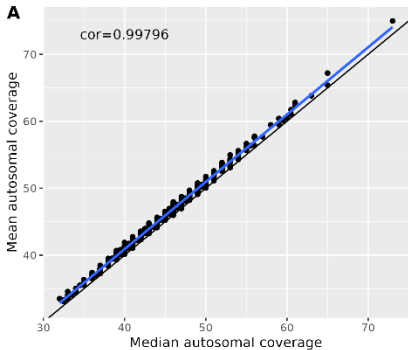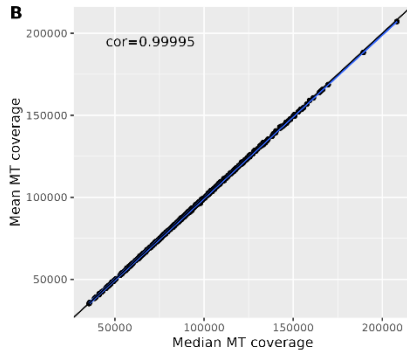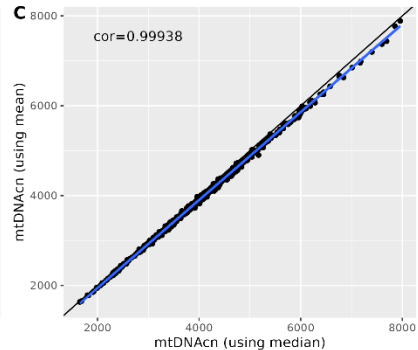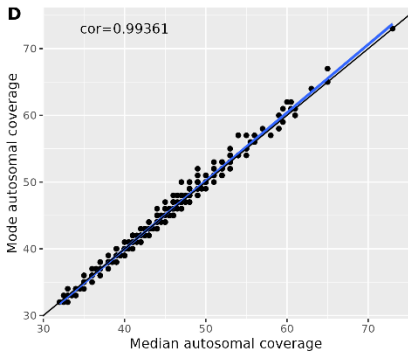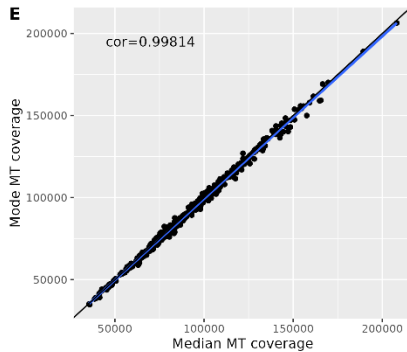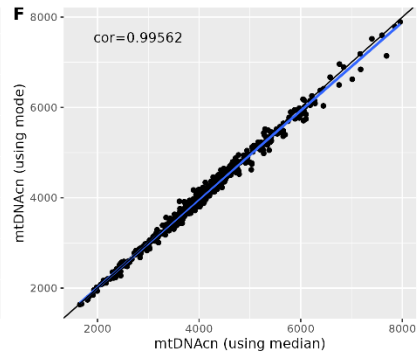

Supplement: S9 Fig — Each dot represents one of the 455 ROSMAP samples from the dorsolateral prefrontal cortex (DLPFC). The black line indicates the diagonal and the blue line represents a linear regression line. The Pearson correlation is displayed in the top left corner. Scatterplots (A–C) depict (A) autosomal coverage, (B) MT coverage, and (C) mtDNAcn using the median (x-axis) versus the mean (y-axis). Scatterplots (D–F) depict (D) autosomal coverage, (E) MT coverage, and (F) mtDNAcn using the median (x-axis) versus the mode (y-axis). The data underlying this figure can be found in S1 Data. (PDF) [file pbio.3002723.s015.pdf]

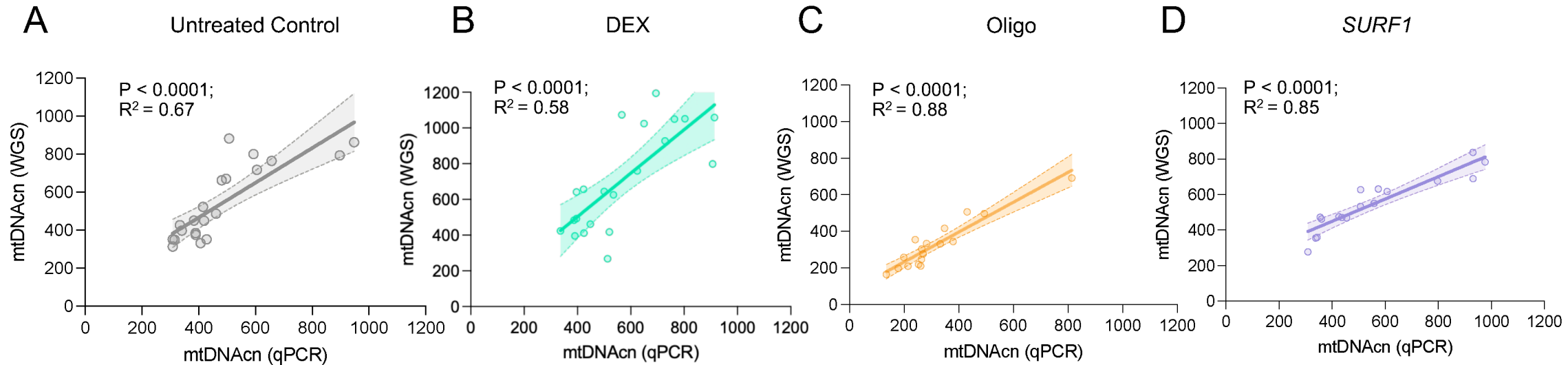

Supplement: S10 Fig — Three donors in each group are merged for analysis. R-squared values and p-values are calculated using standard linear regression models. The data underlying this figure can be found in S1 Data. (PDF) [file pbio.3002723.s016.pdf]
