# Supplementary material for: Somatic nuclear mitochondrial DNA insertions are prevalent in the human brain and accumulate over time in fibroblasts
Source: PLoS Biol. 2024 Aug 22;22(8):e3002723. doi: 10.1371/journal.pbio.3002723 (PMC11340991; doi:10.1371/journal.pbio.3002723)
Supplement: S5 Fig — (A) Numts shared between cell lines (donors) are not significantly correlated with aging. (B) Slopes from cell line-specific Numts and shared Numts in the lifespan model. The data underlying this figure can be found in S1 Data. (PDF) [file pbio.3002723.s011.pdf]

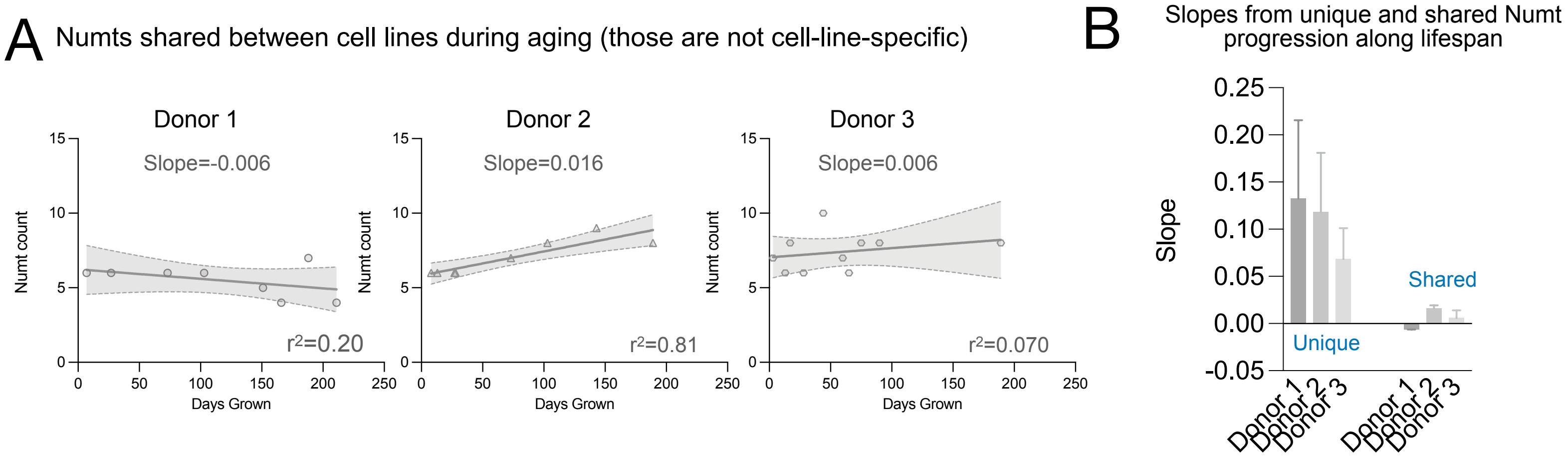
A
Numts shared between cell lines during aging (those are not cell-line-specific)
Donor 1
Slope=-0.006
r^2=0.20
Numt count
Days Grown
Donor 2
Slope=0.016
r^2=0.81
Numt count
Days Grown
Donor 3
Slope=0.006
r^2=0.070
Numt count
Days Grown
B
Slopes from unique and shared Numt progression along lifespan
Slope
Unique
Shared
Donor 1
Donor 2
Donor 3
Donor 1
Donor 2
Donor 3
