# Supplementary material for: Somatic nuclear mitochondrial DNA insertions are prevalent in the human brain and accumulate over time in fibroblasts
Source: PLoS Biol. 2024 Aug 22;22(8):e3002723. doi: 10.1371/journal.pbio.3002723 (PMC11340991; doi:10.1371/journal.pbio.3002723)
Supplement: Attachment — Submitted filename: Reponse_To_Reviewers.Freeze.May7th.docx [file pbio.3002723.s018.docx]

We thank the reviewers for their thorough and constructive comments. We provide our responses below in red and indicate changes made to the primary manuscript in blue.

**Reviewer #1**, Martin Breuss (note, reviewer 1 has signed this review): In this manuscript by Zhou, Karan, and colleagues, the authors describe their analysis of somatic nuclear mitochondrial DNA insertions (Numts) in existing data from human tissues and cell lines. In line with current thinking from many laboratories interested in somatic mosaicism, the authors take a described mutational phenomenon in cancer mosaicism and interrogate its 'normal' occurrence independent of tumorigenesis. Therefore, I believe that the work is timely and interesting, and its description has value for the specific field of somatic mosaicism as well as a broader audience.

However, there are a few aspects that were unclear to me that might necessitate more explanation, different visualization of the data, or changes in their analyses.

We thank Dr. Breuss for his interest and critical assessment of our work.

Major concerns:

1. I would like to see a bit more information on their 'somatic' definition. While I agree with their overall logic, I was missing a more detailed description of their somatic variants. Based on my understanding of the data (and their schematic in Figure 1A) it appears to me that any Numt has to be evidenced by multiple reads to obtain the length of those that go beyond one read. If that is correct, these Numts are likely clonal mosaic events evidenced in multiple cells and originating from development. Could the authors clarify this point?

The reviewer is correct in that our detected Numts in the ROSMAP cohort are likely clonal mosaic events in specific tissues. We are limited to the sequencing data available in ROSMAP, which unfortunately has only ~45X coverage on average, thus extremely limiting our ability to identify low VAF somatic events within individual tissues. Instead, our strategy was to explore Numt accumulation *en masse* on a ‘per tissue’ rather than ‘per individual’ level across the cohort and to use large sets of known transmitted Numts to filter out potential germline events. While we cannot guarantee that any one particular detected Numt is somatic, we clearly observe an enrichment of Numts present in DLPFC and PCC tissues relative to other brain regions and blood independent of potential batch effects that were considered. We have now clarified this in the manuscript text.

Results

Somatic Numt integration differs by tissue, age, and cognitive status (1st paragraph)

We note that the sequencing coverage of these samples was too low (~45X, **Supplementary Table 1**) to confidently identify lower-level somatic mosaicism within any individual tissue sample, though some would still likely be detected by our approach. Our strategy instead was to examine whether there were any potential clonal mosaic events occurring predominantly in one or more tissues compared to others by filtering out all common germline Numts, with the expectation that any rare germline Numts that were not filtered out would be present at a constant rate across all tissues, age ranges, and cognitive categories and thus any differences we observe between them would be driven by somatic events.

- I was missing a description of the observed variant allelic fractions (VAFs) in the text or their VCFs based on how many reads evidenced an event. The only mention of VAFs I could find was in Supp. Figure 8.
- Especially a comparison to known germline events might allow the authors to define mosaicism not only by tissue-to-tissue comparison but potentially by their abundance in the data.

This is an excellent idea. Our expectations were that calculating VAFs for individual tissue samples may be less informative given that we are likely only observing higher frequency clonal mosaic events. However, we agree that a comparison of VAFs between germline and potential somatic events could provide additional scrutiny for lower VAF calls. We have updated our analysis in the **Supp. Fig. 8**, showing the VAF distributions of germline and potential somatic Numts in two datasets and observing that our putative somatic Numts do indeed group towards the lower end of the VAF spectrum relative to germline. We have now included a description describing our relative read support frequencies for each detected call in the ROSMAP cohort as well as in the lifespan-cultured cells.

Materials and Methods

Detection of non-reference Numts

The variant allele frequency (VAF) of each Numt call was calculated based on the number of supporting reads reported by Dinumt divided by the number of overall read coverage in the sequenced genome.

Discussion

Consistent with the ROSMAP tissue data suggesting the accumulation of somatic Numts detected at very low variant allele frequency (VAF) in genomic material (median=4.4%, mean=5.3% ± 3.5%, with 99.7% of total number VAF<35%, **Supplementary Fig. 8**), the variant allele frequency distribution for somatic Numts in fibroblasts was comparably low (median=9.1%, mean=13.1% ± 7.4%, with 98.3% of total number VAF<35%, **Supplementary Fig. 8**).

Supplementary Materials

**Supplementary Figure 8. Variant allele frequency of non-reference Numts in ROSMAP and lifespan study.** The Numts were categorized into germline ones (red), which overlapped with 1KG and Wei et al. callset and shared between tissues (ROSMAP, left) or cell-lines (lifespan study, right), and potential somatic ones (green), which are tissue-specific (ROSMAP, left) or cell-line-specific (lifespan study, right).

2. As this is used quite a bit in Figure 2, I was surprised that the authors did not include a categorical comparison of the disease and age groups for Numt counts. I appreciate that some of this can be deduced from the other figures, but it is not intuitive for a reader.

- Related to age groups: The authors state that they were roughly defined by picking equally sized groups (i.e., doing a separation by thirds). However, the numbers are 295, 319, and 545. This does not appear to be defined by group size, leaving me wondering how the age groups were actually defined by the authors.

The ages of the samples we used from ROSMAP were not uniformly distributed and exhibit a more Gaussian pattern with a mean age of death of 89 years (+/- 6.5 std dev). As such, we opted to use percentiles (30, 40, 30) to delineate the ages into three distinct groups with roughly similar sizes, though this was primarily for visualization purposes. We have now clarified this point in the Methods section. In addition, we added a new supplementary table for the summary statistic numbers in different age groups (age at death) and cognitive status across main tissues in ROSMAP.

Materials and Methods

ROSMAP Cohort

*Data processing*

Based on the clinical information of age, we also stratified all 1187 samples into three age groups with a roughly similar sample size based on their percentiles (30%ile, 40%ile, 30%ile) when fit to a Gaussian distribution: a) samples died at an age younger than 85, b) age at death older than or equal to 85, and less than 93, c) age at death older than or equal to 93 (**New Supplementary Table 2)**.

Supplementary Materials

**New Supplementary Table 2.** Sample counts in different age groups (age at death) and cognitive status across main tissues in ROSMAP.

3. I am unconvinced that using PBMCs with their relatively low sample size is a good/useful baseline for comparison (e.g., Figure 1C). Similarly, it might be better to remove samples with very low sample sizes from the main analyses (as is done for 1D, for instance).

This is a fair criticism, and we have now removed PBMCs from the main analysis, though we still feel it is useful to report our initial findings in these samples (e.g. **Figure 1B**). We now use the cerebellum samples as the control sample for the Hedge’s g calculation in **Figure 1C**.

Results

Somatic Numt integration differs by tissue, age, and cognitive status (4th paragraph)

Relative to the cerebellum, DLPFC and anterior caudate also showed the highest effect sizes (Hedge’s g=1.03 and 1.38, respectively) in Numt abundance, followed by PCC (g=0.49) (**Fig. 1C**).

**Updated Figure 1C:**

**Figure 1**. (C) Effect size (Hedge’s g) of average tissue-specific Numts relative to cerebellum.

4. The authors use Student's t-test for the data presented in Figures 1C and D. In my mind, this should be an ANOVA or comparable test, as they compare all groups to each other without a prior hypothesis. This is further complicated by the vast differences in sample sizes which might complicate some of the standard assumptions for these tests.

We thank the reviewer for this suggestion and agree that a one-way ANOVA test would be more appropriate for our comparisons. We have now rerun our analysis and observed similar results. We have updated the Results section accordingly.

Results

Somatic Numt integration differs by tissue, age, and cognitive status (4th paragraph)

Interestingly, we found that the length of tissue-specific Numts in three brain regions (DLPFC, cerebellum, and PCC) were significantly longer than those observed in whole-blood (median=63bp, One-way ANOVA, p-value<0.001), with PCC-specific Numts themselves exhibiting larger lengths (median=2,477bp) than DLPFC and cerebellum (median=152bp and 210bp, One-way ANOVA, p-value<0.001, **Fig. 1D**).

5. For Figure 1E, as the calling of Numts relies on the mappability of the companion read, could the differences be explained by a systematic bias in identifying Numts in intergenic regions? I saw in the methods for lifespan some mention of GC content and repeat sequence, but I am unsure whether this was taken into account for the analysis in 1E.

We agree that this is certainly a possible explanation for our observations. Our approach, *dinumt*, filters out reads with a MAPQ<10, which could result in false negative results in regions with low mappability. We note that other studies have observed similar trends for both Numts (pmid: 36198798) and mobile element insertions (pmid: 35212372), which could also be impacted by this. We have added this caveat into the main text.

Results

Somatic Numt integration differs by tissue, age, and cognitive status (4th paragraph)

Surprisingly, we found that our somatic Numts integrated into introns at a significantly higher rate compared to its overall expected proportion of the genome based on Ensembl gene annotations (44.58% v.s. 34.92%, p-value<0.001), while a negative enrichment was observed in intergenic regions (53.58% v.s. 63.92%, p-value<0.001, **Methods, Fig. 1E**), though these could be the result of differences of sequence mappability within these regions precluding the detection of Numts.

6. Related to my first comment and relevant for the analyses presented in Figure 2: the negative correlation for DLPFC (especially in NCI) is intriguing. However, the implications may be different depending on whether these events likely arise in development and are found in neurons/neuroglia or are unique private somatic events. Based on the blood data, I would assume that they are likely not derived from infiltrating immune cells but could still be found in microglia. Do the authors have any thoughts on this? I was surprised that the findings of Figure 2 were barely mentioned in the Discussion section.

We agree that a lower rate of Numts in blood cells suggests that DLPFC Numts accumulate later in development and that brain Numts are unlikely to be contributed by infiltrating immune cells. Without cell type specific DNA sequencing data in a large number of individuals, it is not possible to make a conclusive statement about which cell (sub)types contain the Numts. We agree that Numts could be found in microglia. As mentioned in the discussion, the primary human fibroblast data where Numts continue to accumulate at a similar rate even when cells exhibit almost no division (rate of cell division drops by >95% in late life compared to early life) seems to indicate two related points: i) that many private somatic events since same events not always detected in later timepoints, and ii) that cell division is not a major driver of Numts, making it possible that non-replicating neurons and neuroglia alike could be the source of brain Numts. In addition, we added some text in the Discussion section.

Discussion (2nd paragraph)

This observation aligns with the results in cortical brain tissue (DLPFC), a post-mitotic tissue where cell division is expected to be minimal to absent.

7. It is unclear to me from reading the manuscript and methods what the definition of 'cell-line(donor)-specific' vs 'whole-genome overall' Numts is. The difference appears to be small for Numts but large for the deletions. This should be better described, as I am currently not sure what to do with this information.

We apologize for being inexplicit here. Cell-line(donor)-specific Numts referred exclusively to those Numts identified in a unique set of healthy donors or patients with treatment. The terminology 'whole-genome overall' Numts represents 'Total non-reference' Numts identified in these samples and refers to Numts detected at each sequenced individual data point, which can include both germline and potential somatic Numts. However, we agree that including the curves of ‘total non-reference’ Numt here introduced confusion to the readers and was out of the scope of this figure. Thus, we have reworked **Figures 3B, D** along with point #9.

**Updated Figure 3B, D:**

**Figure 3. Numts accumulate in human fibroblasts during normal aging.** (B) Cell-line-specific Numts accumulate over time in aging fibroblasts obtained from three healthy donors cultured up to 211 days. (D) Time-course of cell-line specific deletions in the three healthy donors.

Minor concerns:

8. I am confused by their sentence on lines 145-147: The study cited in reference 35 has a very specific study design to enrich for neocortical variants. Thus, if the authors wanted to highlight the increase in Numts in neocortex vs. the cerebellum, this study is likely not a very good benchmark.

We appreciate the reviewer’s expertise in this area and have chosen to remove this statement from the manuscript.

~~These results are comparable in tissue specificity with recent studies of human brain mosaicism for SNVs and other variant types (35).~~

9. The variability in axes scaling (e.g., Figure 2, Figure 3 for 'lefts' and 'rights') makes it harder for the reader to compare different data.

We agree that our choice of axes labels does make it difficult to directly compare counts between the different categories (**Fig. 2**) or cell line donors (**Fig. 3**). We have now adjusted these to make them consistent.

**Updated Figure 2:**

**Updated Figure 3B, D:** see above.

10. I am unsure why Figure 4D appears to be the only one that does not show individual data points or a box and whisker plot unlike most other figures.

**Figure 4D** is a bar plot to show the number of Numt per 10 days on treatment, representing the slope of Numt accumulation in the groups of patients or healthy donors with or without treatment. The number is derived by 10*$\beta_{treatment}$ of the model below:

$Y_{normalized number of numts} = \beta_{treatment}X_{days} +\beta_{0} + \epsilon$.

As such, each bar represents a single value. The error bars represent the 2.5% and 97.5% confidence intervals for the model parameters.

11. I disagree with the use of the word 'colonization' on line 293 in this context. It implies an active or selected process that benefits the colonizing entity, which I believe is not adequate here.

We have now changed this to ‘accumulation’.

Discussion

Here, we provide some of the first evidence of the somatic nuclear accumulation of Numts in both healthy and impaired human brain tissue across different age ranges.

**Reviewer #2**: This is an excellent paper on a generally underappreciated phenomenon. One wonders if the fact that NUMTs are seen a "contaminants" in mitochondrial sequencing works has lead to a negative view of them and an under-appreciation that these serve as mutational fragments that can litter our nuclear genomes. In fact, NUMT insertions have been found to be the cause of (non-mitochondrial related) genetic disease (and I would suggest the authors highlight this a bit more in the discussion - see below).

From a technical perspective, I have no real concerns. The analysis using the dinumt pathway has been robustly tested elsewhere. Their tissue analysis was quite straightforward. With the sampling limitations better matching was not possible, as clearly stated in the text (which is very appreciated). Limitations to some conclusions due to sample size are clearly stated for the readers, and this conservative, careful approach is appreciated in our current era of "over hyping" of research results. The clear limitations section in the discussions is well done, and as one who also struggles for get "the right" tissue samples for analysis, I can understand the sampling limitations the authors faced.

My only negative comments are editorial in origin, and can be addressed without any further experimentation or analysis.

We thank the reviewer for their appreciation of our work and helpful suggestions.

Major comments;

1. This paper is going to be important for non-neurologists. As such, I would suggest the brain region and other acronyms be kept to a minimum (excluding figures, where the acronym can be spelled out in the legend). Even though the acronyms are spelled out at the start of the results, it is very difficult for a non-specialist (like myself) to follow these unfamiliar short forms (DLPFC, PCC, AC, PBMC, etc).

We appreciate the comment regarding the difficulty in reading articles with a plethora of acronyms. However, given our repeated focus on these brain regions, it would be unwieldy to spell them out at every location throughout the manuscript. To help clarify these terms, we have now updated **Figure 1A** to show the abbreviations and the specific brain regions they refer to, and describe all acronyms in full in each figure legend.

**Updated Figure 1A:**

**Figure 1. Characteristics of tissue-specific Numts in post-mortem human brain regions and blood.** DLPFC, dorsolateral prefrontal cortex; PCC, posterior cingulate cortex. NCI, no cognitive impairment; MCI, mild cognitive impairment; AD, Alzheimer’s dementia.

**Figure 2. Numt association with Age at death by tissue and cognitive impairment.** DLPFC, dorsolateral prefrontal cortex; PCC, posterior cingulate cortex. NCI, no cognitive impairment; MCI, mild cognitive impairment; AD, Alzheimer’s dementia.

2. The only part of the manuscript I have trouble with is the discussion on page 15, where the authors discuss the fact that numts may have functional meaning (lines 329 to 348). Contained within Reference 8 of their manuscript, citations 32-37 list diseases where the genetic lesion or mutation contains a NUMT. I strongly suggest that this be entered in to this argument, as these are clear examples of a functional problem due to a numt insertion.

We thank the reviewer for this suggestion. We hesitate to imply too strongly that these are causal for these disorders, as it is still unclear at this stage whether the mechanism of Numt integration is passive (e.g., through double stranded break repair, which is probably likely) or has some other more active mechanism that forces the insertion into the genome, thus disrupting normal function. However, we agree that it is certainly worth noting the possibility and have now added these references and corresponding text to emphasize the potential functional role of Numts in disease.

Discussion (3rd paragraph)

In contrast, tumors frequently contain Numts within genes, and Numts may even contribute to oncogenesis, which in this case would drive positive selection in tumors(4). Numts have also been implicated previously in several diverse disorders (61-66).

3. Line 33 - I believe here they are talking about the population distribution of numts? If so, the evolutionary constraint infers functional consequence, and this functional consequence point should be more explicitly. Even tied into the disease cases that I mention above.

We agree that evolutionary constraint infers functional consequences and that this likely plays a role in somatic Numt formation and retainment throughout development, however we do not have any specific data or evidence to show this other than their increased presence in some brain regions. We have clarified this by changing ‘evolutionary’ with ‘functional’ in order to indicate that we are primarily referring to potential selective forces during development as opposed to population-level evolution. We have also included the disease references, as noted above.

Discussion (3rd paragraph)

First, strong evidence that detectable Numts are excluded from coding DNA sequences and instead preferentially integrate within intergenic regions (4,6–8) suggest that they are under some functional constraint during development.

4. Line 334 - I am not sure whey they are trying to link Numts moving from the mitochondria and inserting into the nucleus to mitochondrial disease. NUMTs will lead to insertions, which are potentially damaging to the site they insert into. So selection against the MELAS 3243 allele is not really relevant to nuclear DNA insertion mutations. If there are parallels to CNV variation, or other rearrangement mutations in the blood it would be relevant, but selection on heteroplasmic variants has little correlation to nuc-DNA insertions.

We thank the reviewer for this comment. We were not trying to directly link heteroplasmy and Numt integration per se, but instead were noting the parallels between pre- and post-mitotic tissues and their different selective pressures which seems applicable given the lower number of somatic Numt insertions in blood relative to DLPFC, consistent with the lower and higher reported rates of heteroplasmy in those tissues, respectively. In both cases, there is likely a functional pressure regulating these rates and thus we felt it was relevant to note in our discussion.

5. Lines 344-345 - An insert of any size is likely to disrupt a protein sequence, or could disrupt a promoter, etc. So I am not clear on the logic that the size of the insert will lead to "more likely deleterious effects". Can this be spelled out more clearly or omitted?

This is a fair point and we thank the reviewer for pointing this out. There are likely some instances where large insertions may indirectly alter gene regulatory networks (e.g., by altering optimal enhancer distance), but such instances have never been reported for Numts and as such we agree that this should be omitted and it has now been removed.

Discussion (3rd paragraph)

~~Third, and relatedly, larger Numts that due to their size are more likely to be deleterious are observed across multiple post-mitotic brain regions compared to blood immune cells. Blood cells contain Numts approximately 1-2 orders of magnitude smaller than in brain samples, again arguing that selection may occur in replicative tissues, unlike in largely post-mitotic brain cells.~~

Minor points.

6. Line 33 - NUMT would be singular and NUMTs plural? So should it be Numts are…. on this line?

We thank the reviewer for pointing this out. We were referring to the ‘increase’ in the Numts rather than the Numts as a group. However, we can see the confusion and have now reworded this sentence.

Abstract

Moreover, an increase in the number of brain Numts was linked to earlier mortality.

7. In the discussion of NUMTs across species, the authors appeared to mis a few well documented extreme examples, such as the extreme arrays of recent numt inserts in the domestic cat (PMID: 37820546, PMID: 7932781, PMID: 17660503) and the very high percent of the genome made up on numt sequences in the honeybee (PMID: 17383971) and the possum (<https://www.frontiersin.org/articles/10.3389/fevo.2022.844443/full>).

We apologize for these omissions and have now included them in the main text.

Introduction

These nuclear mitochondrial insertions, referred to as ‘Numts’, have been observed in the germline of both human (6,8–11) and non-human (7,12–22) species.

8. In your analysis of new NUMTS - did there happen to be haplogroup informative sequences on the mtDNA sequence that may match the mtDNA of the person and the origins of the NUMTS in any of the reported novel spontaneous insertions?

This is an interesting suggestion. We have now explored this by using the listed haplogroups from ROSMAP. We examined our supporting Numt sequences and found that out of 3,557 somatic Numts we detected in ROSMAP, only 299 (8.4%) were identified as having sufficient signals (≥2 SNPs) to infer haplogroup information. Among these, 266 Numts (89.0% of the 299) matched the reported individual haplogroup information detailed in **Supplementary Table 1**. While this is encouraging, as we would expect most of the somatic Numts to match their parent mtDNA in each sample, the higher rates of mtDNA heteroplasmy in some of these tissues coupled with the limitations of short-read sequencing technology in determining haplotypes makes us hesitant to include this as a formal observation in the manuscript.

**Reviewer #3**: In this paper, Zho et al. study age-related patterns in NUMT insertions in the human genome. They show provide evidence of ongoing numtogenesis (insertion of NUMTs in the nuclear genome) in somatic tissues with time. I think they present important empirical evidence supporting this that has been missing from the literature and I am overall convinced of this result if I don't think too hard about what they are doing. Therein lies my issue: The methods are sometimes unclear and the use of statistical terminology is very loose in certain places, which sometimes makes it difficult to follow exactly what they are doing. I will highlight some of these problems below:

1. L147: Could mtDNA copy number estimation be affected by NUMT count? Could that lead to the association observed?

In the ROSMAP cohort, there is indeed a higher mtDNA copy number in DLPFC than in other tissues (median DLPFC=4047.2 copies per cell, cerebellum=999.4, PCC=4042.2, whole blood=172.0, PBMCs=125.4). We report this in the main text. When we look within each tissue, we do observe a significant correlation between the mtDNA copy number and somatic Numts among the DLPFC samples, yet not in other tissues. In DLPFC, mtDNAcn accounts for 14.6% of the variance in Numts count. We did not observe any correlation between mtDNA copy number and age at death (**Supplementary Fig. 3**).

When comparing the average number of Numts and the mtDNAcn across tissues we observed inconsistent observations regarding the association. In the fibroblast lifespan model, the mtDNA copy number is significantly correlated with the cell-line-specific Numts in the Dex and oligo-treated cell lines. We do not observe this correlation in the untreated groups (i.e. three control cell lines and three SURF1 defect cell lines). In addition, the mtDNA copy number is significantly correlated with the days cultured in all groups (**Supplementary Fig. 7**).

While this could potentially be biasing our results (due to the excess of mtDNA containing sequences that could potentially lead to mapping artifacts), our observations in other tissues with lower detected Numt abundance did not reflect this. Indeed, our observations could be explained by a biological mass action mechanism whereby more mtDNA copies per cell results in more opportunities and potential transfers to the nuclear genome. This is an interesting area that future research could elucidate further, and this is noted as a discussion point in the main manuscript.

2. I don't understand what the percentages in L163 mean. For example, is 45%, the percentage of NUMTs that were discovered within introns? Then, how is 34.92% defined? Also, I don't understand the phrase "compared to its overall composition rate of the genome". Please be clear about the metric being analyzed.

We apologize for not describing this adequately. We used the Ensembel gene annotations (described in the Methods/Genomic content analysis and functional annotation section) to determine the proportion of the genome corresponding to these different categories (e.g. “exonic”, “intronic”, etc, Fig. 1E “Expected”) and then compared this to what proportion of our detected Numts fell within each region (Fig. 1E “Observed”). We have now reworded this section to use “proportion” rather than “composition” to better describe our approach.

Results

Somatic Numt integration differs by tissue, age, and cognitive status (5th paragraph)

Surprisingly, we found that our somatic Numts integrated into introns at a significantly higher rate compared to its overall expected proportion of the genome based on Ensembl gene annotations (44.58% v.s. 34.92%, p-value<0.001), while a negative enrichment was observed in intergenic regions (53.58% v.s. 63.92%, p-value<0.001, **Methods, Fig. 1E**), though these could be the result of differences of sequence mappability within these regions precluding the detection of Numts.

3. L165-166: "significant differences in genic distribution were further observed across the various tissues". Here "genic distribution" is a nebulous term and Fig. 1E or its caption are not clear about this either. For example, the y-axis in Fig. 1E is not defined and the caption defines this as "genic distribution". I have no idea, mathematically speaking, what this is.

We thank the reviewer for this comment and agree that we should have been more specific. The Y-axis is the ‘The percentage of genic and intergenic in the whole genome’ that we have mentioned in the left panel of **Figure 1E**. To be more explicit to the readers, we have broken the original **Figure 1E** into two panels (now the new **Figure 1E, F**) and added a Y-axis title as well as provide more details in the figure legend.

**New Figure 1E, F:**

**Figure 1**. (E) Genic and intergenic distribution of all tissue-specific Numts versus the expected distribution in the whole genome for all samples. (F) Percentage of genic and intergenic distribution of tissue-specific Numts delineated by brain regions and whole-blood.

4. L166: "moderate gene tolerance and potential negative selection of somatic Numt integration in the human brain and blood cells". These two things seem contradictory and I'm not sure how this conclusion follows from the observation that introns are enriched and intergenic regions are depleted of NUMTs compared to the genomic background.

We thank the reviewer for noting this inconsistency. We have revised our wording to better reflect our observations and inferences.

Results

Somatic Numt integration differs by tissue, age, and cognitive status (5th paragraph)

Consistent significant differences in genic distribution were further observed across the various tissues (**Fig. 1F**), suggesting a potential negative selection of somatic Numt integration in the human brain and blood cells, consistent with recent studies (4).

5. L171: "permuting the positions of our observed NUMTs 50,000 times. Permuted with respect to what? The methods weren't clear about this either.

We apologize for not clearly outlining our strategy. Our approach was to take our set of observed Numts and randomly shuffle their positions across the mappable genome 50,000 times and to use that to construct a null distribution from which to test whether our observed categories were significantly deviant. We have now updated the text to better reflect this.

Results

Somatic Numt integration differs by tissue, age, and cognitive status (6th paragraph)

We tested this hypothesis by iteratively assigning random positions for each of our observed Numts 50,000 times across the human genome reference and assessing whether our observed integration sites differed significantly when compared within predefined 10Mb windows across the genome as a permutation test.

Materials and Methods

Genomic analyses for non-reference Numts

*Numt hotspots across chromosomes*

We performed a permutation analysis by randomly shuffling the genomic positions of each observed Numt 50,000 times to determine any hotspots across genome bins compared to the real data.

6. L174: "we observed no significant deviations from random for any of the tested tissues". Significant deviations in what metric? They refer to Fig. 1F, which shows a z-score on the y-axis and the caption does not describe what this z-score represents.

We apologize for not being explicit here. We observed no significant difference between the real Numt distribution and the simulated Numt distribution in the context of different tissues, cognitive impairment levels, and age groups. We reworded ‘deviations’ to ‘difference’ to avoid misunderstanding. In addition, we added the context in the Methods part to explain the Z-score and a detailed title of the y-axis in Figure 1F (now the updated **Figure 1G**).

Results

Somatic Numt integration differs by tissue, age, and cognitive status (6th paragraph)

After the multiple test correction (Benjamini-Hochberg Procedure), we observed no significant difference from random for any of the tested tissues (**Fig. 1G**).

Materials and Methods

Genomic analyses for non-reference Numts

*Numt hotspots across chromosomes*

A Z-score is calculated for normalizing Numt count, which measures the deviation of the Numt count in each 10Mbp bin from the genome-wide average across all the bins.

**Updated Figure 1G:**

7. L179: "paucity of Numts overall may be underpowered" - power is defined for a statistical test. I think they mean to say that the paucity of NUMTS may lead to their permutation test being underpowered?

We thank the reviewer for the correction and have now updated the text.

Results

Somatic Numt integration differs by tissue, age, and cognitive status (4th paragraph)

This is in agreement with previous studies that suggest Numt integration is a random occurrence (6,7,11), though we note that the paucity of Numts may lead to the permutation test being underpowered and thus preclude an accurate assessment at such broad regions across the genome.

8. L187: "each tissue in aggregate" - presumably they mean analyzing all NUMTs in aggregate across tissues.

We thank the reviewer for this correction and have now incorporated this into the main text.

Results

Somatic Numt integration differs by tissue, age, and cognitive status (2nd paragraph)

The remaining 3,713 Numts were then examined in aggregate across tissues to determine whether there were differences in Numt abundance between specific tissues.

9. L201-202: "pathogenicity of AD is likely uncoupled from age-dependent Numt integration". I think this conclusion is coming from the observation that the correlation between NUMT count and age was not statistically significant in AD patients but was significant in the other cohorts. But I don't think they can make this claim without formally testing the slopes across cohorts. The absence of significance in AD patients could be because of lack of power. It does not suggest that there is no age-dependent NUMT integration in AD patients.

We agree and have now softened our statement, though we still feel that this possibility arises from our results and is worth noting as a primer for future studies.

Results

DLPFC-specific Numts are negatively associated with age at death in persons without cognitive impairment

These results indicate that Numts are negatively associated with age at death in certain brain regions of non-AD individuals, suggesting the possibility that brain numtogenesis is deleterious and that the pathogenicity of AD may be uncoupled from age-dependent Numt integration.

10. L245: "somatic Numts could be driven by the simple clonal expansion of few Numts-containing cells". This confused me because throughout the paper I've been thinking of the response variable as being the number of NUMTs observed, not their frequency (depth of NUMT containing reads/total depth at insertion site). I can see how clonal expansion might increase the frequency of existing NUMTs but not how it would lead to an increase in the number of NUMTs.

The reviewer’s perspective on our results is correct as we did indeed focus primarily on the prevalence of Numts rather than their depth of support, which our lower sequencing coverage precludes though we do observe a markedly different difference between putative somatic and germline Numts (Supplementary Fig. 8). However, given the focus in the first part of this project on potential clonal mosaic events in brain tissue, we wanted to explore this in more detail using the lifespan cell culture data, where the results seem to suggest that many of the Numts we are detecting are likely non-clonal.

We also note that while we are not analyzing frequency/depth per se, our ability to detect somatic Numts is still dependent on their underlying frequency. Specifically, a low-frequency Numt that exists in an earlier time point of the cell culture may be undetectable then due to lack of coverage, but if it has clonally expanded over time its frequency may increase to the point that it is detectable at a later time point. As such, we felt it was worth ruling out as a possibility within the constraints of our experiments.

11. L519: "Normalized the NUMT count". It sounds like this is done for each individual. What was the normalization?

12. L520: "Values of Numt Numbers were normalized by the median of Numt counts in each category." This further confused me. Is this referring to the individual-specific normalization discussed in L519 or an additional step?

We have grouped these comments together as they are related. We agree that the description of our normalized methodology was incomplete and have now reworded for clarity.

Materials and Methods

Statistical analysis

Normalized Numts counts were derived by normalizing by the median of Numt counts in each group as defined above for various donor/patient and treatment status combinations.

13. L532: Why was 10Mb chosen to bin the genome?

Given the paucity of these variants, this was an arbitrary cut-off set empirically such that the average genomic bin contained approximately 10 Numts. We have now included this rationale in the manuscript.

Materials and Methods

Genomic analyses for non-reference Numts

*Numt hotspots across chromosomes*

We delineated the entire nuclear genome into 10 Mbp bins, resulting in an average of 10 detected Numts per bin. The frequency for tissue-specific Numts from ROSMAP in each bin was calculated.

14. L548-L550: "test the significant difference between Numt genic distribution and reference genic distribution" - What is the exact metric being compared? What is "reference genic distribution"? Does "reference" imply genome background/non-genic regions?

We used Pearson’s chi-square test and Fisher’s exact test to assess the statistical significance of the discrepancies between the observed and expected distributions of Numts within genic or intergenic regions. These tests compare 2x2 categorical variables, specifically the proportion of Numts within a particular genomic region against the one in the rest of the genome, with respect to either the observed or expected data. We have expanded the text in the Methods part to be more explicit about it.

Materials and Methods

Genomic analyses for non-reference Numts

*Genomic content analysis and functional annotation*

Pearson’s chi-square test and Fisher’s exact test were used to assess the statistical significance of the discrepancies between the observed and expected distributions of Numts within genic or intergenic regions. We compared 2x2 categorical variables, specifically the proportion of Numts within a particular genomic region against the one in the rest of the genome, with respect to either the observed or expected data.

Other questions:

15. What commands were used to generate autosomal and mtDNA coverage? Samtools? Was any filtering involved?

We used the R/Bioconductor (packages GenomicAlignments and GenomicRanges) to calculate the sequence coverages of the autosomal chromosomes *covnuc* and of the mitochondrial genome *covmt*. And then mtDNAcn was calculated using autosomal coverage with the following formula: mtDNAcn = (covMT / covautosomal) × 2. We only used the reads with MAPQ>0 to conduct the calculations. The above analysis has been published in Hans-Ulrich Klein et al. *Mol. Neurodegener.* 2021 (PMID: 34742335) and was cited properly in our manuscript. Further, R scripts can also be found here: github.com/cu-ctcn/mtDNA/blob/main/mtDNAcn/calculateMtDNAcn.R. We have described the filter in detail and attached the GitHub page to our manuscript.

Materials and Methods

Genomic analyses for non-reference Numts

We calculated the mtDNAcn using autosomal coverage with the following formula: mtDNAcn = (cov_MT_ / cov_autosomal_) × 2 (60) in both the ROSMAP dataset and lifespan study. The median sequence coverages of the autosomal chromosomes *covnuc* and of the mitochondrial genome *covmt* were calculated using R/Bioconductor (packages GenomicAlignments and GenomicRanges). We filtered out the reads with MAPQ=0 in the analysis.

R and shell scripts used for mtDNA analysis are deposited at GitHub: https://github.com/cu-ctcn/mtDNA (60).

In conclusion, the paper adds value to the scientific literature, but the authors need to be clearer about their methods.

We again thank the reviewer for their critical assessment and have hopefully now clarified our methodology and approaches.
