# Supplementary material for: Somatic nuclear mitochondrial DNA insertions are prevalent in the human brain and accumulate over time in fibroblasts
Source: PLoS Biol. 2024 Aug 22;22(8):e3002723. doi: 10.1371/journal.pbio.3002723 (PMC11340991; doi:10.1371/journal.pbio.3002723)
Supplement: Attachment — Submitted filename: Reponse_To_Reviewers.Revision2.docx [file pbio.3002723.s019.docx]

We thank the reviewers for their comments and their additional suggestions for improvements. We provide our responses below in red and indicate changes made to the primary manuscript in blue.

**Reviewer #1**, Martin Breuss (note, reviewer 1 has signed this review): The authors have addressed all my concerns and no additional major concerns arose from their response to other reivewers.

**Reviewer #2:** All of the comments I made are addressed to my satisfaction. They reply to point 8, while not explicitly publishable, is very encouraging that the observed treands in the NUMTs are true novel insertions. I appeciate that the authors did this, and replied so candiidly.

We are pleased we were able to address these concerns and again thank the reviewers for their helpful comments.

**Reviewer #3:** I thank the authors for approaching the comments constructively and taking the time to respond. I have some minor suggestions:

We thank the reviewer and appreciate their efforts to help us improve and clarify our work

1. In response to point 2 of mine, the authors have described clearly what the different percentages refer to: "the proportion of our detected NUMTs that fell within each region". This is more explicit than the "rate of NUMT insertion" as phrased in the main text and I encourage them to use the former language for clarity.

We have now included this specific language in the main text, as well as replacing ‘rate’ with ‘frequency’ to be more explicit.

**Results**

**Somatic Numt integration differs by tissue, age, and cognitive status**

We focused on the Numts that were inserted in and around transcribed regions of the genome (introns, coding, UTR, and intergenic regions) and examined the proportion of our detected NUMTs that fell within each region. Surprisingly, we found that our somatic Numts integrated into introns at a significantly higher frequency compared to its overall expected proportion of the genome based on Ensembl gene annotations (44.58% v.s. 34.92%, p-value<0.001), while a negative enrichment was observed in intergenic regions (53.58% v.s. 63.92%, p-value<0.001, Methods, Fig. 1E), though these could be the result of differences of sequence mappability within these regions precluding the detection of Numts.

2. Re: point 4, the revised text is still vague and not saying much. Presumably, the evidence for negative selection in a particular tissue would be a depletion of NUMT insertion into coding or UTR regions compared to background expectation. This was not observed (Fig. 1F). Instead there were significant differences in NUMT enrichment in intronic and intergenic regions that the authors interpret to be due to mapping artifacts. Yet they continue on to say that "consistent significant differences in genic distribution were further observed across the various tissues, suggesting a potential negative selection of somatic NUMT integration in the human brain and blood cells" in Line 179 of the revised text. How are they able to draw this conclusion? Which aspect of the genic distribution are they referring to? What direction of difference is expected in the presence of negative selection? Do they see that?

This is a fair point given the way we designed these analyses (as addressed below), and as such have removed the remark regarding selection from the text and now only note that these patterns were consistent across tissues.

These significant differences in genomic distributions were further observed across the various tissues (**Fig. 1F**).

3. Re: point 8, the revised text in the response document is correct but in Line 200 of the revised text, the authors still say "We first examined each tissue in aggregate and observed almost no correlation in NUMT abundance with the age of death". They mean to say: "We first examined NUMTs in aggregate across tissues observed no correlation in NUMT abundance with the age of death".

We thank the reviewer for catching this and have now updated the text accordingly.

**Results**

**DLPFC-specific Numts are negatively associated with age at death in persons without cognitive impairment**

We first examined NUMTs in aggregate across tissues and observed almost no correlation in Numt abundance with the age of death (Fig. 2A).

4. In Line 183 of the revised text, the authors say they hypothesized that the genomic distribution of somatic NUMT integration sites may differ between tissues or across age groups. They tested this hypothesis by permuting NUMT positions 50,000 times across the genome and assessing whether the distribution of integration sites (proportion of NUMTs within each 10Mb bin) differed significantly from a uniform distribution (based on permutations). This was done separately for each tissue type and age group. This is a minor technical point but this test addresses the question: Is NUMT integration within tissue X (or within age group X) distributed uniformly at random? It does not explicitly address the question of whether NUMT integration differs across tissues or age groups as stated in the main text. They did not find any significant differences so this does not ultimately matter but might be useful to make that distinction for clarity.

The reviewer brings up a valid point and we thank them for their rigor. We have now modified the text to more accurately reflect our analysis.

**Results**

**Somatic Numt integration differs by tissue, age, and cognitive status**

We lastly hypothesized that the genomic distribution of somatic Numt integration sites might differ within individual tissues, cognitive status, or age groups from an expected random distribution throughout the genome.
